# Voluntary-Driven Elbow Orthosis with Speed-Controlled Tremor Suppression

**DOI:** 10.3389/fbioe.2016.00029

**Published:** 2016-03-31

**Authors:** Gil Herrnstadt, Carlo Menon

**Affiliations:** ^1^MENRVA Lab, Engineering Science Department, Simon Fraser University, Burnaby, BC, Canada

**Keywords:** exoskeleton, elbow orthosis, assistive robot, tremor suppression, force control, simulation, voluntary motion

## Abstract

Robotic technology is gradually becoming commonplace in the medical sector and in the service of patients. Medical conditions that have benefited from significant technological development include stroke, for which rehabilitation with robotic devices is administered, and surgery assisted by robots. Robotic devices have also been proposed for assistance of movement disorders. Pathological tremor, among the most common movement disorders, is one such example. In practice, the dissemination and availability of tremor suppression robotic systems has been limited. Devices in the marketplace tend to either be non-ambulatory or to target specific functions, such as eating and drinking. We have developed a one degree-of-freedom (DOF) elbow orthosis that could be worn by an individual with tremor. A speed-controlled, voluntary-driven suppression approach is implemented with the orthosis. Typically tremor suppression methods estimate the tremor component of the signal and produce a canceling counterpart signal. The suggested approach instead estimates the voluntary component of the motion. A controller then actuates the orthosis based on the voluntary signal, while simultaneously rejecting the tremorous motion. In this work, we tested the suppressive orthosis using a one DOF robotic system that simulates the human arm. The suggested suppression approach does not require a model of the human arm. Moreover, the human input along with the orthosis forearm gravitational forces, of non-linear nature, are considered as part of the disturbance to the suppression system. Therefore, the suppression system can be modeled linearly. Nevertheless, the orthosis forearm gravitational forces can be compensated by the suppression system. The electromechanical design of the orthosis is presented, and data from an essential tremor patient is used as the human input. Velocity tracking results demonstrate an RMS error of 0.31 rad/s, and a power spectral density shows a reduction of the tremor signal by 99.8%, while the intentional component power was reduced by <1%.

## Introduction

Among movement disorders known to affect humans, pathological tremor is highly prevalent (Elble and Deuschl, 2011); estimates show it to affect above 10% of the elderly population (Wenning et al., 2005; Shahed and Jankovic, 2007; Tse et al., 2008; Barbosa et al., 2013). Tremor has been defined as an “approximately rhythmic, roughly sinusoidal involuntary movement” (Elble, 2009). In addition to its high prevalence, multiple classifications and conditions of tremor exist. In fact, more than 10 clinical subtypes of tremor have been defined in the medical literature, with the most common being essential tremor (ET) and Parkinson’s disease (PD) (Elble and Deuschl, 2011). Tremor often affects the upper extremities (Wyne, 2005), and can adversely impact the ability to perform basic tasks. Additionally, social discomfort is a common grievance with tremor patients (Louis, 2001). No known cure is available and medication, albeit not always effective, comprises the primary course of therapy (Rocon and Belda-Lois, 2004; Deuschl et al., 2011).

Over the past few decades, a variety of mechanical-based solutions have been proposed for lessening patients’ tremors, and in so doing assisting them in their activities of daily living (ADL). Devices for tremor suppression can be categorized as passive or active as well as ambulatory, typically as wearable devices, or non-ambulatory (Rocon and Belda-Lois, 2004; Rocon et al., 2007b). Non-ambulatory devices can nevertheless serve users in their ADL (e.g., by being connected to a wheelchair). More typically, non-ambulatory devices are grounded (e.g., connected to a table) for use in a home or a clinic.

Early investigations into biomechanically suppressing tremor resulted in devices that were predominantly non-ambulatory (Hendriks et al., 1991; Aisen et al., 1993); additionally, these devices relied on damping forces. Several wearable (ambulatory) assistive devices have also been suggested since that target the upper limbs, wrist, and elbow joints (Kotovsky and Rosen, 1998; Loureiro and Belda-Lois, 2005; Rocon et al., 2007a; Ando and Watanabe, 2012). The suppressing technologies employed ranged from passive damping to active damping, and to the use of actuators such as electric motors. In contrast to the aforementioned investigative devices, only a limited number of devices offering passive and active tremor attenuation have become commercially available (Michaelis, 1988; Pathak et al., 2014), and are for the most part non-ambulatory in nature.

Functional electrical stimulation (FES) and soft actuators, based on conducting polymer polypyrrole and piezoelectric fiber composites and polymer films, for tremor suppression have been suggested by several research groups as an alternative to systems using rigid components and actuators (Skaarup et al., 2007; Swallow and Siores, 2009; Popović Maneski et al., 2011; Zhang et al., 2011; Dosen et al., 2014). Electromyographic (EMG) sensors are often utilized in conjunction with FES. Recognized limitations for FES include muscles redundancy and coupling involved in the activations of joints, surface electrodes hardware limitation in accessing specific muscles, and muscle fatigue.

Impedance control strategies have been suggested by multiple researchers (Pledgie et al., 2000; Rocon et al., 2007a; Taheri et al., 2011). The impedance control approach attempts to modify the human–machine frequency response such that higher impedance is present at the tremor frequencies (Taheri et al., 2011). The main drawback of impedance control involves sensitivity to inaccuracies in the human–machine model parameters or changes thereof over time.

Distinguishing the tremor and voluntary components from a recorded signal is a fundamental step in tremor applications, whether for diagnosis or treatment. Online signal decomposition in particular poses a greater challenge than an offline computation. Strategies ranging from linear filtering to stochastic estimators have been employed. Gonzalez et al. (2000) designed an optimal digital filter offline through pursuit tracking tasks. Ando and Watanabe (2012) used a second-order low-pass filter (LPF) applied to an EMG tremor signal to be passed on to a neural network, intended to control an elbow device. Verstappen et al. (2012) used a high-pass filter to separate the tremor component before passing it to a repetitive control loop using an FES system. Another recent work utilized a tremor estimator in the form of a high-pass filter (Taheri et al., 2014). The filter resulted in a significant phase shift, which was corrected prior to being applied to the suppressive actuator. The inherent phase shift of linear filters is considered their main limitation (Rocon and Belda-Lois, 2004).

Another estimation method is the weighted-frequency Fourier linear combiner (WFLC). The WFLC adaptively models a tremor signal by tracking its frequency, amplitude, and phase (Riviere and Thakor, 1996; Riviere et al., 1998). However, for best performance, pre-filtering with a high-pass filter is recommended. Several variations of the WFLC method have been proposed (BMFLC and ASBMFLC) since that can adaptively adjust the frequency bandwidth for the tremor estimation (Veluvolu et al., 2010; Wang et al., 2014). A different approach, the adaptive band-pass filter (ABPF), was proposed by Popović et al. (2010) and compared favorably to the WFLC.

The Kalman filter (KF) is a stochastic estimator, based on a Bayesian model, traditionally employed in navigation (e.g., satellite) and ballistic tracking (Kalman and Bucy, 1961). The KF has been utilized for tremor suppression by several researchers. Rocon et al. (2007a) and Rocon and Pons (2011) have implemented a KF to track the voluntary motion and by subtracting it from the total motion, obtain an estimation of the tremor. The tremor signal was used to control a three degree-of-freedom (DOF) upper arm orthosis. Additionally, g-h and Benedict–Bordner filters were employed. The main difference between the above filters is the method of weights selection (Brookner, 1998). Widjaja et al. (2008) have also implemented the KF, fusing information from accelerometer and EMG data, to obtain a single joint (one DOF) estimate of the tremor angle to be used in diagnosis, classification, and FES applications.

It is not uncommon to assess tremor suppression methods by performing simulations, either numerically or experimentally (Hashemi et al., 2004; Popović et al., 2010; Veluvolu and Ang, 2011; Zhang et al., 2011; Taheri et al., 2012, 2015; Chuanasa and Songschon, 2014; Wang et al., 2014) and obtaining access (with ethics consent) to patients once simulations have yielded optimal results. Simulations can promote tuning and debugging of the suppression system, and thus, improve the performance. Furthermore, recordings from patients can be used to simulate the tremor profile helping to bridge the gap between simulations and testing with subjects.

Most strategies to suppress tremor employ and rely on inertial sensors. In this work instead, the sensing of interaction forces between the user and the suppression system is the primary feedback measurement used. Sections “Suppression Approach” and “Orthosis System” detail the suppression approach and the orthosis system. The controller implementation is covered in Section “Orthosis Control” and the testing procedure in Section “Testing Procedure.” The results and conclusion are presented in Sections “Results” and “Discussion.”

## Materials and Methods

### Suppression Approach

The approach presented in this article aims to guide a tremor suppression device to follow the voluntary motion of a user having tremor. The device must suppress the tremor by resisting the respective motion, while simultaneously moving along with the voluntary motion.

**Figure 1:**
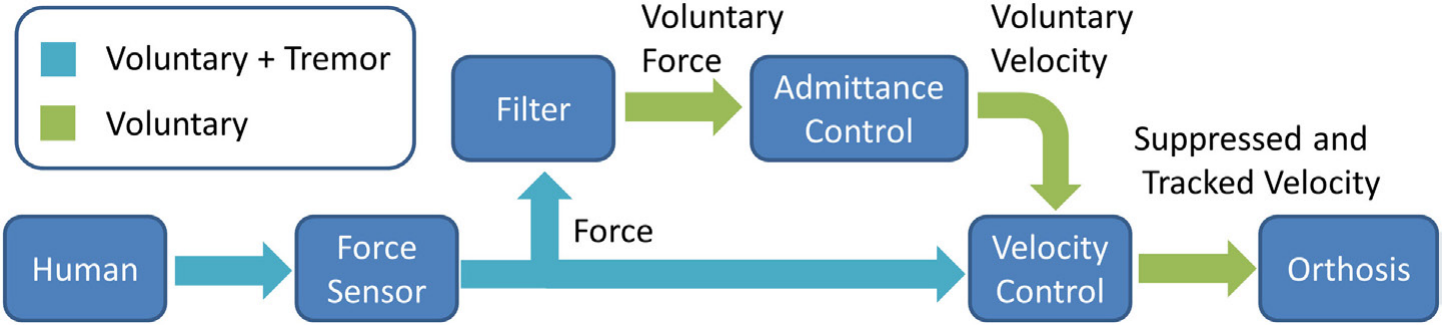
**Suppression approach elementary blocks**.

The tremor suppression approach responsible for tracking the voluntary motion of a human can be described using its elementary components as shown in the block diagram of Figure 1. A force transducer measures the mechanical interaction forces between a human and the suppression device. The sensed force includes both the voluntary and tremor components, as does the human motion. Therefore, the subsequent “Filter” block separates the voluntary from the total force signal. Most commonly, works dealing with tremor suppression utilize primarily the tremor signal, following its decomposition from the total recorded signal, in the suppression approach. Instead, this work primarily makes use of the voluntary component signal. One of the basic premises of almost any tremor suppression approach, used also in this work, is that tremor frequencies occupy a different frequency range than most daily voluntary motions (Rocon and Belda-Lois, 2004). The aforementioned is key to successfully decomposing a tremorous motion. Once the voluntary force has been isolated with the filter block, it is passed on to the admittance control block. The admittance controller, typically defined as (Zeng and Hemami, 1997)

(1) $$R\left(s\right)=\frac{\dot{X}\left(s\right)}{F\left(s\right)},$$

accepts a force input and outputs a velocity command (upper path in Figure 1). The velocity controller in turn tracks a velocity command representing the voluntary motion received from the admittance controller. From the controller design perspective, this work considers the human tremor as a source of disturbance to the orthosis. The velocity controller acts to reject tremor disturbances that may influence and interfere with the orthosis voluntary velocity motion. Thus, the tremor forces applied by the human (lower path in Figure 1) are suppressed, while the voluntary component is tracked by the orthosis. The suppression approach is illustrated in a generic and modular form in Figure 1 such that each block may be implemented interchangeably with a variety of modules.

### Orthosis System

**Figure 2:**
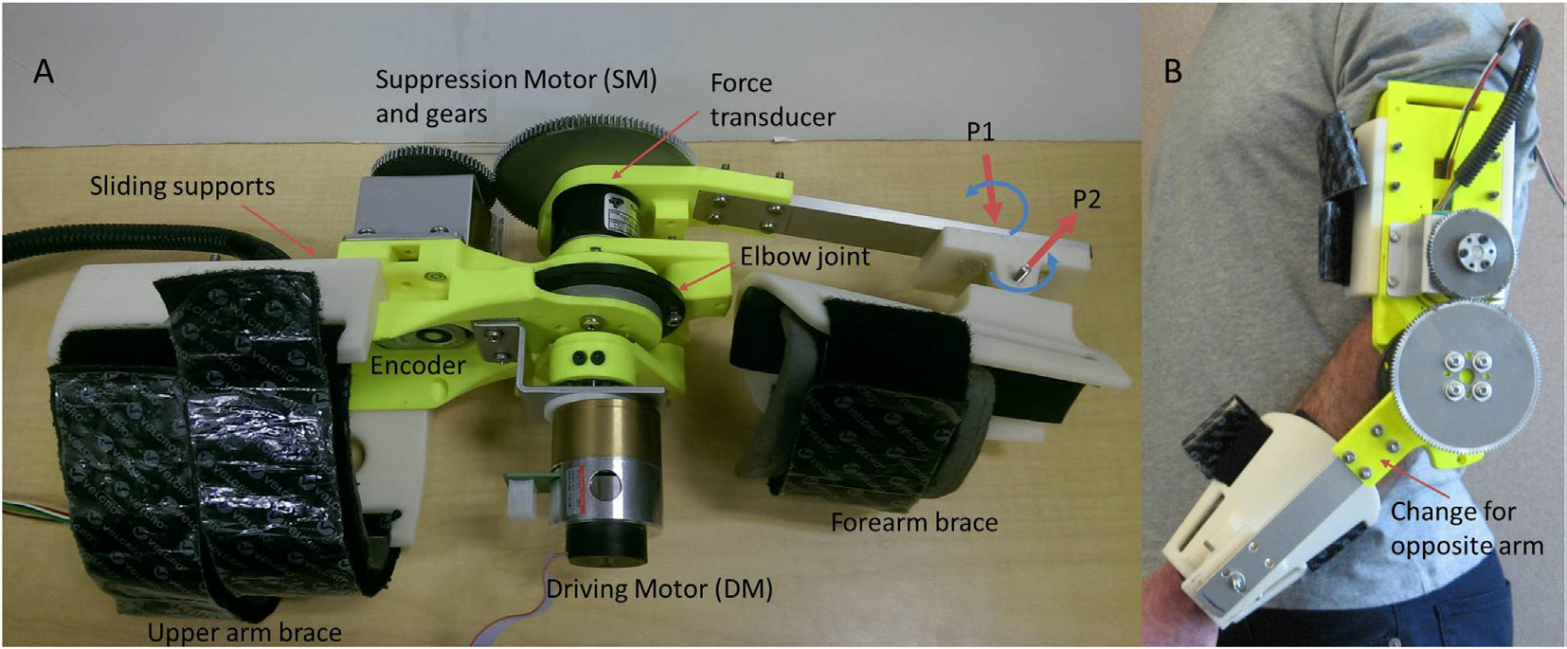
**Orthosis system**. **(A)** The orthosis simulation system connected to the DM. P1 and P2 indicate the two passive wrist joints. **(B)** The orthosis donned.

An orthosis system was developed in order to validate the suggested tremor suppression approach through experimental testing. The orthosis, shown in Figure 2, is a one DOF system targeting the human elbow, composed of a suppression motor (SM), gearing, sensors that include a force transducer and an encoder, and upper and forearm braces. The SM gearing includes an off the shelf spur gearbox with a reduction ratio of 26:1 and additional external spur gears with a reduction ratio of 120:72. The upper and forearm braces as well as the main body of the orthosis were 3D printed using an ABS plastic variant. An aluminum beam is used to connect the forearm brace to the main orthosis body. Several fit adjustments were incorporated into the orthosis design as shown in Figure 2A. Adjustment for upper arm length is achieved through sliding of the top and bottom supports, while adjustment for the forearm is made possible through two passive intersecting joints (P1, P2) in the forearm brace as shown in Figure 2A. The orthosis donned is demonstrated in Figure 2B. The orthosis is all but symmetric. Adjustability for right or left arms is achieved by replacing only a single part (marked in Figure 2B), and switching between the upper arm top and bottom sliding supports.

In this paper, the tests are limited to the evaluation of a test-bench (no individuals with tremor are included) in order to evaluate the feasibility of the proposed approach. For this purpose, a driving motor (DM) is added to the orthosis (see Figure 2A) to simulate the tremorous input that could be provided by a human subject. Details of the orthosis components and performance are provided in Table 1.

**Table 1:** **Orthosis system specifications**.

Components		Performance	

Name	Model number	Criteria	Value
SM	Maxon EC 45 flat P/N 339286	Stall torque (Nm)	16.5
SM gearbox	26:1 Maxon Spur Gearhead GS 45 A P/N 301173	Continuous torque (Nm)	3
DM	Maxon EC 45 flat P/N 339287	Weight (g)^a^	1600
DM gearbox	18:1 Maxon Spur Gearhead GS 45A P/N 301175	Max speed (rpm)	109.1
Force transducer	Transducer Techniques, TRT-100	Elbow range (°)	0–120
Force amplifier	Transducer Techniques, TM0-1		

*^a^DM not included in the measurement*.

### Orthosis Control

**Figure 3:**
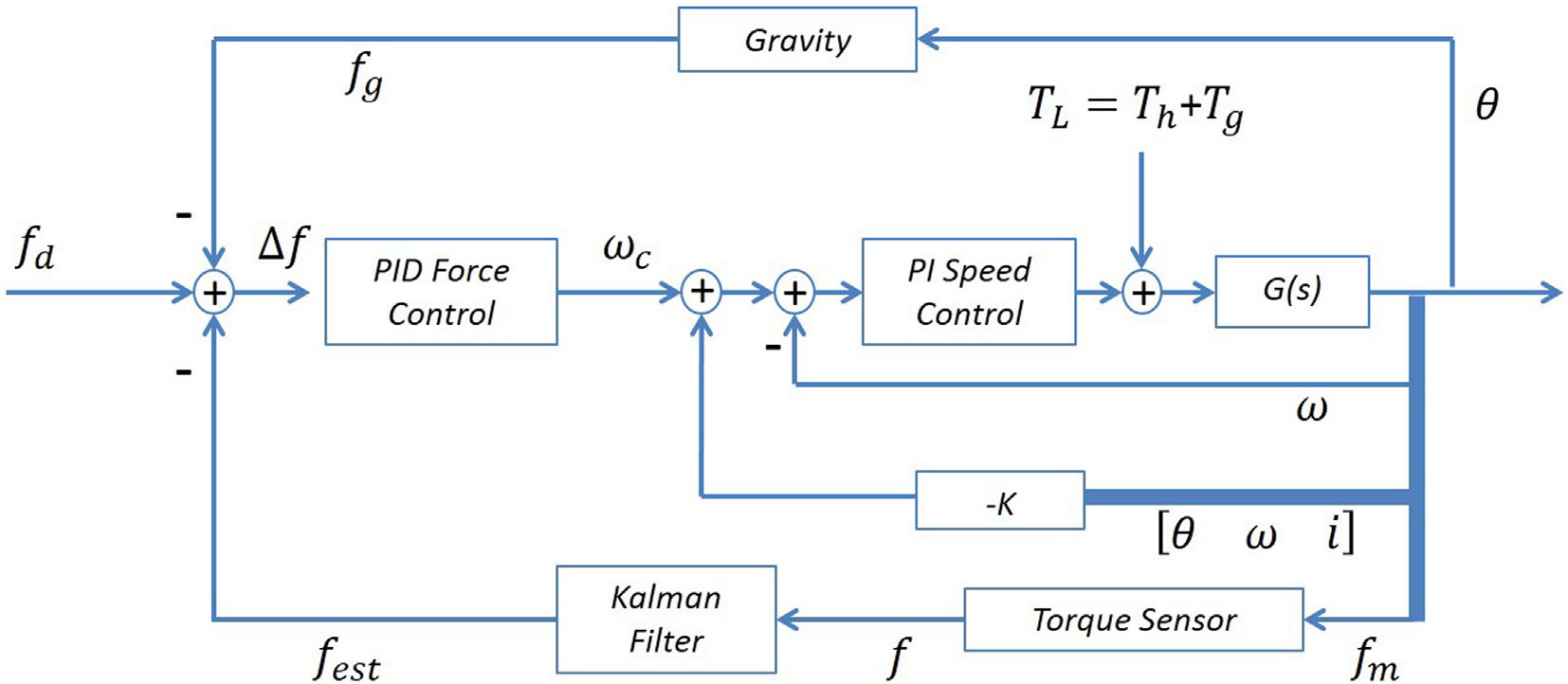
**Orthosis control diagram**. The controller includes a force feedback, with an inner speed controller and state feedback, as well as a gravity compensation loop.

The control of the orthosis was implemented using the feedback loops shown in the block diagram of Figure 3. A PID force controller was used as the admittance block (Vitiello and Lenzi, 2013), and a PI controller was used as the speed controller corresponding to the elementary blocks of the approach presented in Figure 1. The filter block was implemented with a KF, a stochastic estimator that is often employed in both tremor suppression as well as non-tremor-related applications (Kamen and Su, 1999; Gallego et al., 2009). As mentioned in Section “Suppression Approach,” tremor and voluntary motion frequencies typically have little overlap. ET and PD frequencies are considered to be in the range of 4–12 and 4–6 Hz, respectively, while ADL tend to be in the range of 0–2 Hz (Mann et al., 1989; Wyne, 2005; Taheri et al., 2015). The KF regards the tremor component as a stochastic noise and is, therefore, able to distinguish it from the voluntary component. Other filters may be used successfully, such as a g-h filter or a LPF. However, it should be noted that a chosen filter’s performance is expected to influence the overall suppression system performance. *G*(*s*) refers to the SM and gearbox model, which form part of the suppressive system along with the force sensor. A state feedback was also incorporated to improve the speed controller tracking. The system being controlled as in Figure 3 can be shown to be stable (Herrnstadt and Menon, 2015). Several saturations were implemented with both the speed controller, limiting the acceleration/deceleration of the SM (± 23077 rpm/s), and with the force controller, limiting its output velocity into the speed controller (± 115 rpm).

In this work, in addition to the human input labeled *T_h_*, the disturbance to the suppressive system also includes the orthosis forearm gravitational forces (non-linear dynamics) labeled *T_g_*, as shown in Figure 3. The gravity disturbance component due to the orthosis forearm link can be compensated with an additional loop as shown in Figure 3 (top loop) such that the signal *f_g_* and subsequently Δ*f* entering into the admittance controller acts to counteract for the gravity component. The admittance controller can be represented as

(2) $$\omega_{c}=K_{\mathrm{fp}}\Delta f+K_{\mathrm{fd}}\frac{\mathrm{d\Delta f}}{\mathrm{dt}}+K_{\mathrm{fi}}\int_{0}^{t}\mathrm{\Delta fds},$$

where *K_fp_*, *K_fd_*, and *K_fi_* are suitable PID gains, and ω*_c_* is the controller velocity output. The input to the force controller is defined as

(3) $$\Delta f=f_{d}-f_{\mathrm{est}}-f_{g},$$

where *f_est_*, *f_d_*, and *f_g_* are the estimated human voluntary interaction force applied to the orthosis, the desired interaction force and the gravitational force, respectively. It should be noted that the orthosis forearm link physical parameters are predetermined and known; thus, the gravity load is deterministically known and bounded. The gravity compensation is updated online as a function of the measured position (Dung, 2010; Artemiadis, 2014).

The suppression system is intended to be transparent to the user such that when pushed against, the orthosis moves away with minimum resistance [i.e., *f_d_* = 0 in Eq. 3] (Gupta and O’Malley, 2006; Duchaine et al., 2012). Furthermore, it is assumed that the human is capable of performing voluntary motions independently as well as counteracting the gravitational forces acting on their own limb unassisted. Therefore, compensation for the human arm by the suppression system is not incorporated.

### Testing Procedure

This work involved simulating the human input to the system with the DM. The purpose of the simulation is to validate the tremor suppression approach using the orthosis prior to testing with a human subject. Experimental simulations can help to optimize and debug the system, while promoting its safety. Data from an ET patient (Timmer et al., 2000) were used as an input to the DM simulating the human motion. The patient’s raw data used in the experiment were preprocessed to convert it (from linear acceleration units) to angular velocity units. Furthermore, the orthosis forearm gravity force was compensated by the suppression system. The physical parameters of the orthosis forearm were used. The forearm link and cuff, of the prototype fabricated in our lab (Figure 2), have an equivalent moment arm and mass of approximately 117 mm and 290 g, respectively.

The performance of the suppression-on case was evaluated relative to the unsuppressed case, representing the free human tremor without the effect of the suppressive system. The acquisition of the suppression-off signal was done with the DM physically disconnected from the suppression system. It should be noted that the reference velocity signal was obtained offline, from the suppression-off data, for the evaluation of the system and would not be available in a real application with a human user.

The admittance (PID) controller tuning was done heuristically, however, initial controller values can be found based on the Ziegler and Nichols method (Corripio, 2001). The ultimate gain and period procedure can be applied to the closed loop orthosis system. A small perturbation is provided with the DM and the proportional gain is increased until constant oscillations are achieved. The ultimate gain and period can then be extracted in order to obtain the initial controller gains.

As mentioned in Section “Orthosis Control,” the control goal was defined as *f_d_* = 0. However, it is noted that while fine-tuning the controller, the suppression-on velocity signal was also considered relative to the desired reference velocity. Some performance compromise between achieving a small interaction force and close velocity tracking was required. Generally, the derivative gain contributed less to the overall performance, as is often the case in practice (Ang et al., 2005). The state feedback was added once the PID tuning was obtained. The PI controller was pre-tuned by the manufacturer.

A PC and a data acquisition device (NI USB-6341) were used in the experiments to collect and process the signals. Programing and control algorithms were implemented in NI LabVIEW 2013 software. Additionally, MathWorks Matlab R2013b was used for offline processing and for producing plots.

## Results

**Figure 4:**
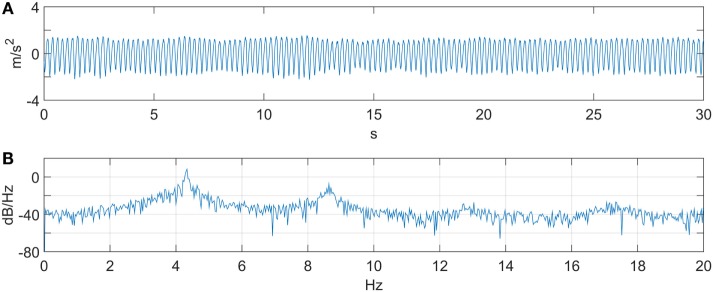
**ET patient data (et02)**. **(A)** Linear acceleration. **(B)** PSD. A first harmonic tremor frequency is observed at 4.3 Hz.

The input provided by the user follows a velocity profile. The velocity input was constructed from data of an ET patient (et02). The original raw data and its Power Spectral Density (PSD) plot are shown in Figure 4. The patient data has a first harmonic tremor frequency at 4.3 Hz. Once the patient data were converted to angular velocity units, they were superimposed with a sinusoidal velocity signal having a frequency of 0.8 Hz and amplitude of 1 rad/s, representing the voluntary motion. The gravity compensation for the orthosis arm was performed by the suppression system as demonstrated in Figure 3. As mentioned in Section “Testing Procedure,” the unsuppressed intentional velocity was acquired in order to serve as a reference for the suppression-on case. A velocity signal was recorded while the DM was physically disengaged from the robot arm and the SM, representing the unsuppressed human motion containing the voluntary and tremor components. Consequently, zero phase filtering of the suppression-off velocity resulted in the voluntary velocity component that would serve as the reference signal. When activated, the suppression system should track the filtered velocity signal associated with the suppression-off case.

**Figure 5:**
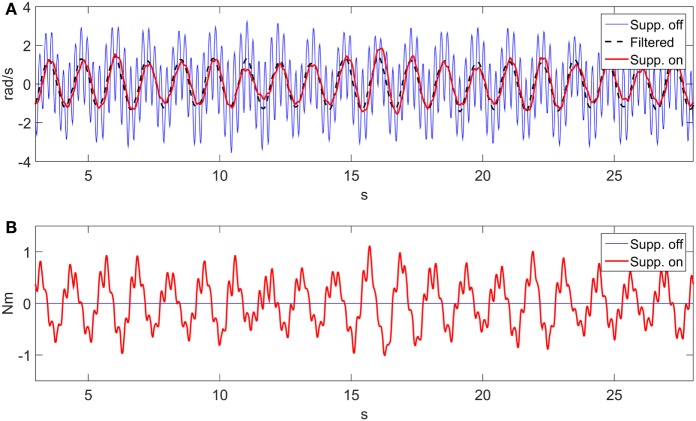
**Suppression velocity and force tracking results**. **(A)** Velocity tracking. **(B)** Interaction force.

**Figure 6:**
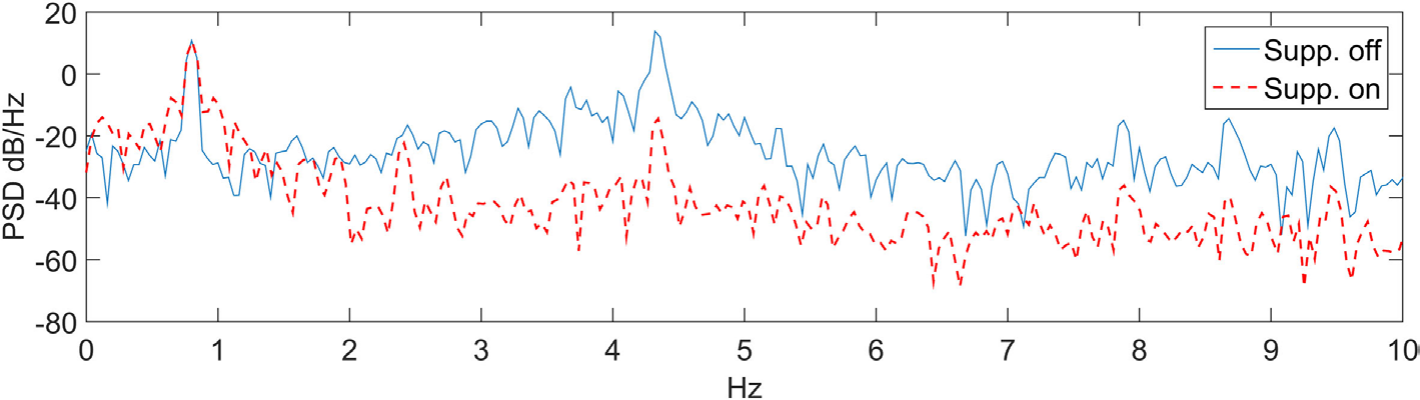
**PSD in the suppression-on and suppression-off cases**.

The velocity tracking and interaction forces obtained are shown in Figure 5. Velocity tracking errors are apparent at the peaks of the sinusoid motion in Figure 5A. The velocity peaks correspond to the interaction force crossing the zero line. The forces, produced by the DM, around the zero crossing are less consistent and consequently are responsible for larger velocity tracking errors. The RMS values of the velocity tracking error and of the interaction force signals are 0.31 rad/s and 0.44 Nm, respectively. The PSD of the suppression-off and suppression-on signals were also compared as shown in Figure 6. A clear power reduction can be observed in the tremor frequencies, particularly above 2 Hz, while in the vicinity of the intentional motion frequency (0.8 Hz) the signals overlap, indicating a small impact to the voluntary component. The tremor power reductions for the first and second harmonics were 99.8 and 99.1%, respectively, and 99.8% combined, while the change to the intentional component power was −0.15%.

## Discussion

The work presented here involves the application of a novel tremor suppression approach to a custom prototype elbow orthosis. Information describing the impact suppression systems have on the intentional component of the motion tends to be limited in tremor suppression-related literature. In this study, the performance with regard to both components of the motion, i.e., the tremorous and the intentional are emphasized. A review of performance metrics to quantify tremor is provided by Rocon and Belda-Lois (2004). In this work, the time domain is evaluated by measuring the velocity and force tracking errors RMS, while the PSD is used to evaluate the spectral domain, similar to other publications in the field. PSD for the voluntary, first and second harmonics were calculated for the 0–2, 2–6, and 6–10 Hz frequency bands, respectively.

The suggested approach considers the human input and the orthosis forearm link as disturbances to the suppression system. This notion carries several benefits. Human arm parameters are not required for the controller tuning. Additionally, the controller design is simplified to a linear system case. Compensating for the gravitational effects on the human arm is also not needed.

The velocity tracking and PSD results indicate that the system can successfully follow the desired motion profile while significantly reducing the tremor. Our studying of the first two tremor harmonics was based on a finding reported by Taheri et al. (2015) where it was shown that the first two harmonics contain the majority (above 94%) of the signal power.

The transfer function associated with Figure 4 can be expressed as a second-order system similar to that provided in Pledgie et al. (2000), Rocon and Belda-Lois (2004), and Taheri et al. (2011), with the addition of a differentiator to obtain a velocity output. The associated transfer function describes the relationship between the human muscle torque and the associated joint output velocity. In our experiments instead, a motor (DM) is simulating the human input. The transfer function describing the implementation of Figure 4 is

$$G_{\text{DM1}}\left(s\right)=\frac{1}{M_{h}s^{2}+C_{h}s+D_{h}}$$

(4) Mh=JLaKgKm,Ch=(JRa+KfLa)KgKm,Dh=KfRa+KbKg2KmKgKm,

where *J, R_a_, L_a_, K_f_, K_m_*, and *K_g_* are the moment of inertia, motor armature resistance, armature inductance, motor damping coefficient, torque constant, and the gear ratio, respectively. *G_DM1_* in Eq. 4 refers to a transfer function from input voltage to output velocity, and has a similar structure to transfer functions in the literature describing a human arm, with *M_h_, C_h_, D_h_* representing the moment of inertia, damping, and stiffness of the arm joint.

**Figure 7:**
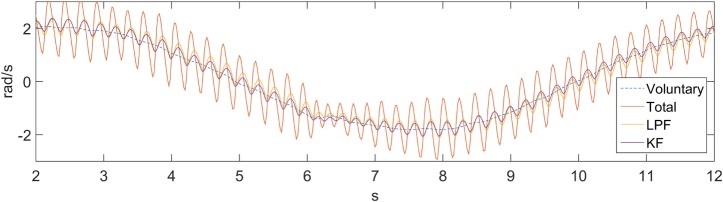
**Kalman filter and a low-pass filter comparison**.

To demonstrate the feasibility of utilizing other filters in the “Filter” block of Figure 1, we performed an offline comparison between a KF and a LPF, shown in Figure 7. A first-order Chebyshev filter with cut-off frequency of 2 Hz and 0.1 dB ripple was considered as a measure of comparison. The difference between each filter (LPF and KF) and the voluntary component was calculated and a RMS was applied to the error. A PSD was also calculated for each filter, and the total signal power (in the 2–6 Hz band) was obtained. The error RMS for the LPF and the KF are 0.21 and 0.13 rad/s respectively. The signal power reduction, relative to the total signal, for the LPF and the KF are 92.1 and 94.8%, respectively. The LPF relatively small performance loss in relation to the KF suggests that a LPF could be used successfully with the proposed suppression approach. Another work comparing between a LPF and the BMFLC with KF is available in Veluvolu and Ang (2011), and demonstrates superior performance to the latter filtering method.

At its foundation, impedance control provides a frequency-dependent relation between motion (i.e., of a limb) and a force (Pledgie et al., 2000; Hashemi et al., 2004; Rocon et al., 2007a; Taheri et al., 2015). The impedance controller can modify the biomechanical characteristics of the system (robot and limb) to achieve attenuation at the tremor frequency. However, it has been shown by several researchers that loading of the tremorous body part (externally or internally) can cause changes to the tremor properties, such as the amplitude and frequency (Joyce and Rack, 1974; Rack and Ross, 1986; Aisen et al., 1993; Héroux et al., 2009). In turn, the impedance relationship between the motion and the force may be altered, thus compromising the effectiveness of an impedance controller.

Adaptive control strategies may help mitigate impedance changes to successfully attenuate tremor, though these may only effectively account for internal loading (i.e., muscle activation). To the authors’ best knowledge, adaptive impedance control has yet to be investigated for the application of tremor suppression. Taheri et al. in their recent work suggested a tremor suppression controller designed to attenuated tremor near the tremor fundamental frequency. The controller adapted online to the tremor frequency (Taheri et al., 2015). Nonetheless, the stiffness, damping, and mass impedance properties did not adaptively update.

Some works unrelated to tremor have investigated adaptive impedance controllers. The idea behind adaptive impedance control is to maintain consistent system performance in the presence of robot or environment parameter uncertainty (Zeng and Hemami, 1997). Learning impedance control has been researched in manipulator control, typically for industrial applications (Buchli et al., 2011). Investigations involving rehabilitation devices have also been carried out. Hussain et al. (2013) developed a control scheme whereby the robot assistance is adapted based on the level of disability or participation expressed by the user. Adaptive impedance has been considered for prosthetic devices in order to achieve more natural capabilities, similar to the human limbs (Perreault et al., 2014). For the application of tremor suppression, biological feedback, such as EMG, may be used to guide the learning of the impedance control law. In the same vein as in the work by Hussain et al. (2013), a change in muscle participation or in the combination of muscles used for a given task may require a change of the controlled impedance.

As mentioned previously, most tremor suppression methods model the tremor signal and generate a corresponding suppressing command. An impedance-controlled system that models the tremor signal may, therefore, experience fluctuation in the suppression performance due to the impedance variability. By contrast, the proposed method only models the voluntary motion. Therefore, variability in the limb impedance is not expected to affect the attenuation performance.

A pertinent question concerning the approach proposed here is whether it is intuitive for a user to have the resulting orthosis velocity be related to the interaction torque. It is conceivable that instead a more intuitive manipulation approach may be to control the orthosis in torque/acceleration, based on the input interaction torque. Additionally, it is important to consider that the orthosis developed in this work can be reduced further in size. However, for the purpose of this study, the presented orthosis was used successfully to validate the suppression approach.

## Conclusion

The steps taken in this work are aimed at demonstrating the feasibility of the approach in reducing pathological tremor, when implemented with an elbow orthosis. We obtained above 99% tremor power reduction while the effect on the voluntary signal power remained below 1%. The system was able to track the desired velocity signal with an RMS error of 0.31 rad/s, while the interaction force RMS was 0.44 Nm. The results obtained with the suggested approach are encouraging and should be validated in future studies with human volunteers having tremor.

## Author Contributions

GH developed system concept, conducted testing, and collected data. Drafted and revised the article. CM contributed guidance and advice throughout the process, including concept development and design, and interpretation of data. Participated in manuscript drafting and has provided approval of the final version to be submitted.

## Conflict of Interest Statement

The authors declare that the research was conducted in the absence of any commercial or financial relationships that could be construed as a potential conflict of interest.
