# Supplementary material for: Voluntary-Driven Elbow Orthosis with Speed-Controlled Tremor Suppression
Source: Front Bioeng Biotechnol. 2016 Mar 31;4:29. doi: 10.3389/fbioe.2016.00029 (PMC4814799; doi:10.3389/fbioe.2016.00029)
Supplement: Supplementary file 1 [file Data_Sheet_1.docx]

# Appendix

In order to implement the KF, a dynamic model for the interaction force signal was used as described in the following evolution equations:

|  | $D_{k+1}=D_{k}+T\tilde{d}_{k}$ | **(5)** |
| --- | --- | --- |

|  | $M_{k+1}=M_{k}+TD_{k}+\frac{1}{2}T^{2}\tilde{m}_{k}$ | **(6)** |
| --- | --- | --- |

where the parameters $M_{k}$ and $D_{k}$ represent the $k^{th}$ iteration measured torque and its first derivative ($D_{k}=\frac{dM_{k}}{dt}$). The KF model controllable input is unknown as it is determined by the human applied force. $T$, $\tilde{m}_{k}$ and $\tilde{d}_{k}$ are the sampling time, the measured force noise and the force-derivative noise respectively. In matrix form the system can be described as follows

|  | $X_{k+1}={AX}_{k}+Gw_{k}$  $y_{k+1}={CX}_{k}+v_{k}$ | **(7)** |
| --- | --- | --- |

where $w_{k}$ and $v_{k}$ are the process and measurement noise respectively. The respective vectors and matrices from **(7)** are defined as

|  | $X_{k}=\left[ \begin{matrix} M_{k} \\ D_{k} \end{matrix} \right], w_{k}=\left[ \begin{matrix} \tilde{m}_{k} \\ \tilde{d}_{k} \end{matrix} \right]$  $A=\left[ \begin{matrix} 1 & T \\ 0 & 1 \end{matrix} \right], G=\left[ \begin{matrix} \frac{T^{2}}{2} & 0 \\ 0 & T \end{matrix} \right], C=\left[ \begin{matrix} 1 & 0 \end{matrix} \right]$ | **(8)** |
| --- | --- | --- |

The process and measurement noise covariance matrices, and the initial state estimation error covariance were defined as (with the assumption that $\tilde{m}_{k}$ and $\tilde{d}_{k}$ are uncorrelated)

|  | $Q=E\left\{ w_{k}w_{k}^{T} \right\}=\left[ \begin{matrix} 4e-6 & 0 \\ 0 & 0.04 \end{matrix} \right]$  $R=E\left\{ v_{k}v_{k}^{T} \right\}=0.0001$  $P_{0}=\left[ \begin{matrix} 1.6e-13 & 0 \\ 0 & 1.6e-5 \end{matrix} \right]$ | **(9)** |
| --- | --- | --- |

where $Q, R, P_{0}$ are the process, measurement noise, and initial estimation error covariance respectively. The initial states $X_{0}$ were set to zero.
